# The Cleavage-Specific Tau 12A12mAb Exerts an Anti-Amyloidogenic Action by Modulating the Endocytic and Bioenergetic Pathways in Alzheimer’s Disease Mouse Model

**DOI:** 10.3390/ijms24119683

**Published:** 2023-06-02

**Authors:** Valentina Latina, Anna Atlante, Francesca Malerba, Federico La Regina, Bijorn Omar Balzamino, Alessandra Micera, Annabella Pignataro, Egidio Stigliano, Sebastiano Cavallaro, Pietro Calissano, Giuseppina Amadoro

**Affiliations:** 1 European Brain Research Institute (EBRI), Viale Regina Elena 295, 00161 Rome, Italy; 2 Institute of Biomembranes, Bioenergetics and Molecular Biotechnologies (IBIOM), National Research Council (CNR), Via Amendola 122/O, 70126 Bari, Italy; 3 Research Laboratories in Ophthalmology, IRCCS—Fondazione Bietti, Via Santo Stefano Rotondo 6, 00184 Rome, Italy; 4 Institute of Translational Pharmacology (IFT), National Research Council (CNR), Via Fosso del Cavaliere 100, 00133 Rome, Italy; 5 Area of Pathology, Department of Woman and Child Health and Public Health, Fondazione Policlinico Universitario A. Gemelli IRCCS, Istituto di Anatomia Patologica, Università Cattolica del Sacro Cuore, Largo Francesco Vito 1, 00168 Rome, Italy; 6 Institute for Biomedical Research and Innovation (IRIB), National Research Council (CNR), Via P. Gaifami 18, 95126 Catania, Italy

**Keywords:** tau immunotherapy, tau cleavage, Amyloid Precursor Protein (APP), Amyloid beta peptide (Aβ), Alzheimer’s Disease (AD), endocytosis, bioenergetics, neuroprotection

## Abstract

Beyond deficits in hippocampal-dependent episodic memory, Alzheimer’s Disease (AD) features sensory impairment in visual cognition consistent with extensive neuropathology in the retina. 12A12 is a monoclonal cleavage specific antibody (mAb) that in vivo selectively neutralizes the AD-relevant, harmful N-terminal 20–22 kDa tau fragment(s) (i.e., NH_2_htau) without affecting the full-length normal protein. When systemically injected into the Tg2576 mouse model overexpressing a mutant form of Amyloid Precursor Protein (APP), APPK670/671L linked to early onset familial AD, this conformation-specific tau mAb successfully reduces the NH_2_htau accumulating both in their brain and retina and, thus, markedly alleviates the phenotype-associated signs. By means of a combined biochemical and metabolic experimental approach, we report that 12A12mAb downregulates the steady state expression levels of APP and Beta-Secretase 1 (BACE-1) and, thus, limits the Amyloid beta (Aβ) production both in the hippocampus and retina from this AD animal model. The local, antibody-mediated anti-amyloidogenic action is paralleled in vivo by coordinated modulation of the endocytic (BIN1, RIN3) and bioenergetic (glycolysis and L-Lactate) pathways. These findings indicate for the first time that similar molecular and metabolic retino-cerebral pathways are modulated in a coordinated fashion in response to 12A12mAb treatment to tackle the neurosensorial Aβ accumulation in AD neurodegeneration.

## 1. Introduction

Clinical, neurophysiopathological and neuroimaging studies have proposed the eye as a direct surrogate for the detection and monitoring of CNS neurodegeneration in vivo [1,2,3,4,5], in particular for Alzheimer’s Disease (AD), the most common cause of dementia in the elderly [6,7,8,9,10]. The age-dependent accumulation of Aβ and hyperphosphorylated/cleaved tau protein, along with other structural and functional synaptic and metabolic changes classically taking place in AD brains, are also detected in the eyes of animal models [2,11,12,13,14,15] and affected patients [4,16,17,18,19,20,21,22,23,24]. Consistently, deficits in visual system function and in vision-dependent cognition occur in AD animal models [25,26] and human subjects [12,27,28,29,30]. Mechanistically, a causal transsynaptic spreading of proinflammatory and amyloidogenic neuropathology has been proposed to develop along the entorhinal-hippocampal-retinal axis throughout the moderate/late stages of AD progression [31,32,33,34,35]. Furthermore, in human beings and in vivo model systems, visual spatial complaints and retinal functional deficits are reported to manifest in concomitance [8,11,36,37] or, sometimes, even precede [38,39] the occurrence of the signs of memory/learning deterioration traditionally associated with the clinical symptomatology of AD. Thus, from a translational point of view, ocular biomarkers’ accuracy and reliability are currently being assessed by noninvasive imaging techniques to facilitate an early AD diagnosis, prognosis evaluation and response to treatment [11,40,41,42].

In this framework, we have previously reported that neurosensory retina and hippocampus in parallel respond to tau-directed intervention in 6-month-old Tg2576, a well-established preclinical AD animal model which only expresses the human Amyloid Precursor Protein (APP) 695 with Swedish mutation (K670N-M671L). In agreement with complementary results from another research group reporting a pharmacological eye-brain positive immunomodulation of Aβ [43], we have demonstrated that selective neutralization of 20–22 kDa toxic tau fragment (i.e., NH_2_htau) following intravenous (i.v.) injection of cleavage-specific 12A12 monoclonal antibody (mAb) in vivo relieves behavioral (deficits in spatial memory and orientation), neuropathological (accumulation of APP/Aβ, tau hyperphosphorylation and truncation) and metabolic (mitochondrial impairment) retinal-cerebral parameters associated with animals’ AD phenotype [15,44,45]. Interestingly, we have also found out that systemic treatment with 12A12mAb exerts a local anti-amyloidogenic effect both in the hippocampus and retina of transgenic AD mice, supporting the finding that a dynamic, positive feed-forward regulation between APP/Aβ and tau cleavage drives the neurodegeneration in this disease [46,47]. However, despite the growing body of evidence advocating the tight physiopathological relationship between the eye and brain, likely due to their similar neuroectodermal origin [1,2,3,4,5], the concomitant retino-cerebral responses to systemic administration of AD-relevant therapeutic antibodies and their potential action mechanism(s) have been poorly investigated in preclinical animal models.

Several lines of evidence have demonstrated that dysregulation of endocytic signaling accompanied by alterations in the intracellular distribution of APP gives rise to the Aβ overproduction/accumulation at synapses of AD patients and animal models, including Tg2576 [23,44,45,46,47,48,49,50,51,52,53,54,55]. The processing/maturation of APP along the amyloidogenic route to yield toxic Beta-secretase-1 Carboxy-Terminal-Fragments (βCTFs)/Aβ is actually an intricate cellular process that is strongly regulated by the endocytic pathways. It involves an initial step in which membrane-bound APP is internalized and converges with Beta-secretase-1 enzyme (BACE1) into Ras-related protein 5 (Rab5)-positive early endosomes where β-cleavage occurs [56,57] followed by its subsequent γ-secretase-dependent maturation in late endosomes/trans-Golgi network to give rise to Aβ [50,58]. More importantly, vesicular trafficking and localization of both BACE1 and APP are coordinated and convergent events [59] since the amyloidogenic processing pathway ends up being strongly enhanced when BACE1 or APP levels concomitantly increase in a common subcellular endocytic compartment [60,61,62,63]. Changes in bioenergetic pathways and in the inverse cellular relationship existing between glycolysis and mitochondrial respiration flux also critically influence the fate of APP processing towards the amyloidogenic route [64].

In view of these literature findings, the anti-amyloidogenic effect exerted in vivo by 12A12mAb both in the brain (hippocampus) and retina was investigated by analyzing key aspects of the endocytic and bioenergetic pathways which control at synapses the BACE1-triggered APP trafficking/maturation towards the amyloidogenic route. By means of a combined biochemical and metabolic experimental approach, the results of this preclinical study elucidate the crucial molecular mechanisms underlying the beneficial action of 12A12mAb in decreasing the Aβ generation in Tg2576 mice.

## 2. Results

### 2.1. 12A12mAb Immunization Antagonizes the BACE1-Initiated Amyloidogenic Processing of APP by Altering the Protein Expression of Neuron-Specific BIN1 and RIN3 Endocytic Adaptors, Both in Hippocampus and Retina from Tg2576 AD Mice

Among the key endosomal trafficking regulator, the Bridging INtegrator 1 (BIN1) protein has been shown to negatively regulate clathrin-mediated endocytosis by preventing APP/BACE1 segregation in early endosomes [65,66]. A knock-down of BIN1 prevents in neurons the transport of BACE1 out of the early endosomes toward the recycling endosomes leading to excessive accumulation/encounter between BACE1 and APP, which eventually gives rise to accelerated Aβ production and accumulation [67,68,69]. Interestingly Genome-Wide Association studies (GWAS) have also described BIN1 as a genetic locus for susceptibility of both Late Onset AD (LOAD) and sporadic Early Onset AD (sEOAD) [69]. Additionally, and more importantly, the proline-rich region into N-terminus projection extremity of tau—which is specifically targeted in vivo by 12A12mAb [15,44]—is capable of binding to the SH3 domain of BIN1 [70,71,72].

**Figure 1:**
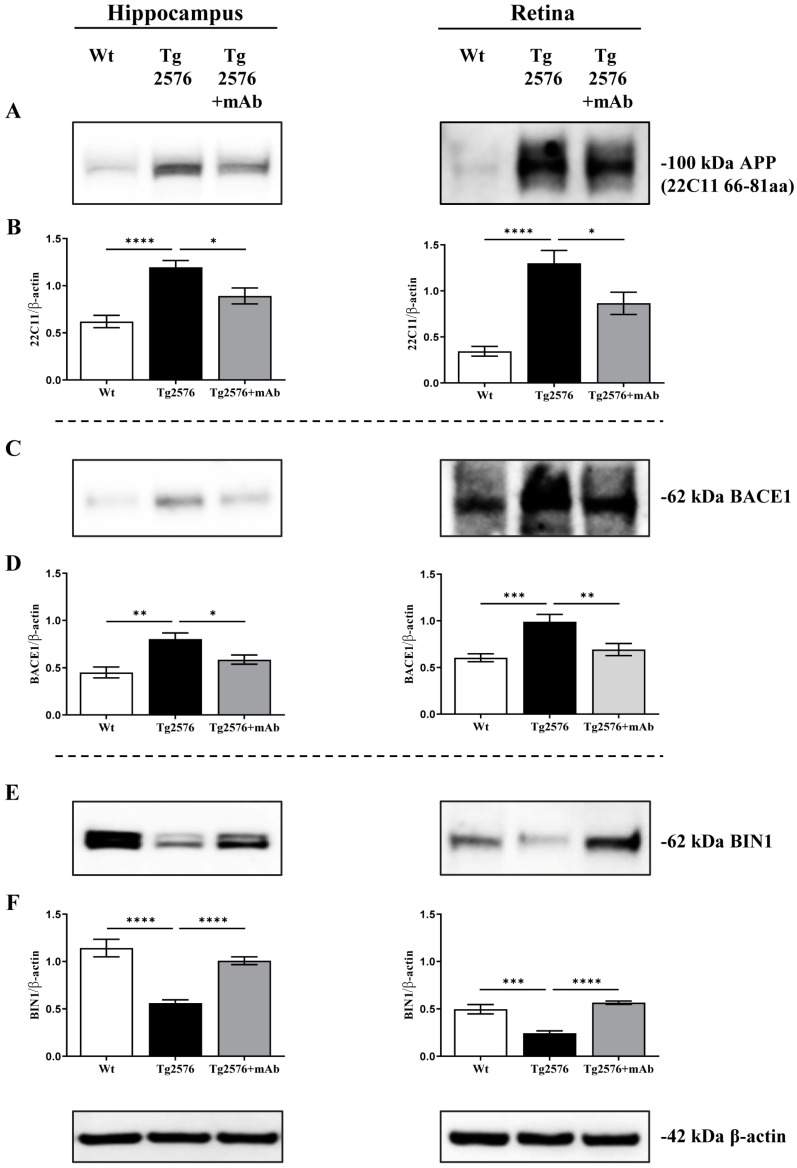
Immunization with 12A12mAb modulates the expression of neuron-specific BIN1, a crucial endocytic regulator governing the dynamic convergence of APP and BACE1 and then the Aβ generation. (**A**,**C**,**E**) Representative images of SDS-PAGE Western blotting analysis (*n* = eight animals per each group, four males and four females for each experimental condition) carried out on synaptosomal preparations of hippocampus and retina from animals of three experimental groups (littermate wild-type, vehicle-treated Tg2576, Tg2576+mAb) with antibodies for APP, BACE1 and BIN1 (as indicated alongside the blots). Dashes on the right side indicate the molecular weight (kDa) of bands calculated from the migration of standard proteins. (**B**,**D**,**F**) Histograms show the semi-quantitative densitometry of the intensity signals of bands by normalization with β-actin level used as a loading control. *p* < 0.05 was accepted as statistically significant (one-way ANOVA followed by Bonferroni’s post hoc test for multiple comparisons among more than two groups * *p* < 0.05; ** *p* < 0.01; *** *p* < 0.0005; **** *p* < 0.0001).

In view of these considerations, to give insights into the molecular mechanisms underlying the anti-amyloidogenic effect of 12A12mAb both into the brain and retina from 6-month-old Tg2576 [15,44], the expression level of BIN1 was examined under our experimental conditions. Western blotting SDS-PAGE analyses followed by semi-quantitative densitometry were carried out on crude synaptosomal preparations of hippocampus and retina from animals of three experimental groups (littermate wild-type, vehicle-treated Tg-AD, Tg-AD+mAb) by probing with specific antibodies for APP (22C11), BACE1 and BIN1 (99D) (Figure 1). Crude synaptosomal fractionation procedure—which includes all endocytic structures (endosome- and clathrin-coated vesicles) [73]—was performed to obtain pure, isolated fractions (Supplementary Figure S1) enriched in neuron-derived synaptic vesicles due to the preferential localization of APP undergoing extensive processing by APP-cleaving enzyme BACE1 secretase in this subcellular compartment [74]. On immunoblot of brain and retina endocytic-enriched extracts, BIN1 appears as multiple bands corresponding to differently spliced 12 isoforms, the largest of which (65 kDa) is only expressed in neurons but not in astrocytes and microglial cells (BIN1.1) [75,76]. As shown in Figure 1E,F and regardless of the analyzed area (hippocampus versus retina), the steady state expression level of neuron-specific BIN1 isoform (65 kDa BIN1.1) was significantly reduced in Tg2576 AD mice when compared with littermate wild-type group (hippocampus **** *p* < 0.0001; retina *** *p* < 0.0005, Tg2576 versus wild-type). This finding is in line with the evidence that downregulation of BIN1 is associated with poorer memory performance in AD cases [77,78,79,80] and in this preclinical AD model, especially in the concomitant presence of tau neuropathology [69,81,82]. Consistent with the local activation of the amyloidogenic pathway with the generation of Aβ specie(s) in these transgenic mice [15,44,83,84,85], the intensity signal of APP (Figure 1A,B) and BACE1 (Figure 1C,D) was inversely increased into synaptic compartments from both tissues (hippocampus **** *p* < 0.0001, ** *p* < 0.01; retina **** *p* < 0.0001, *** *p* < 0.0005; Tg2576 versus wild-type). These results also match well with the upregulation of APP and BACE1 activity and their expression levels detected in AD subjects with high cerebral Aβ load [83,86,87,88,89,90]. Interestingly (Figure 1E,F) and in concomitance with the successful neutralization of toxic NH_2_htau (Supplementary Figure S2) [15,44], we found that 12A12mAb immunization in both tissues significantly prevented the decline in the immunoreactivity signal of BIN1.1 isoform in Tg2576 AD cohort when compared to its not-immunized counterpart (hippocampus **** *p* < 0.0001; retina **** *p* < 0.0001; Tg2576+mAb versus Tg2576). In line with the neuroprotective and anti-amyloidogenic action exerted in vivo by treatment with this tau antibody in parallel in hippocampus and retina as shown by Haematoxylin and Eosin (H/E) staining and Western blotting with specific APP/Aβ antibodies [15,44] (Supplementary Figures S3 and S4, respectively), the synaptic amount of APP and BACE1 was significantly downregulated in 12A12mAb-treated cohort (hippocampus * *p* < 0.05; retina * *p* < 0.05, ** *p* < 0.01; Tg2576+mAb versus Tg2576) (Figure 1A–D).

**Figure 2:**
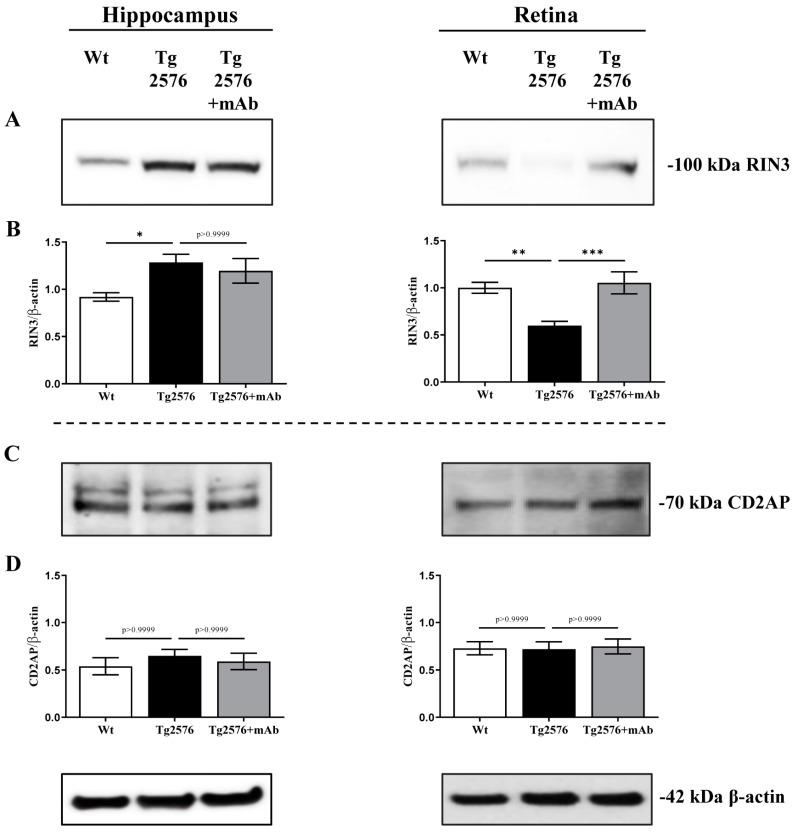
Change in RIN3 but not CD2AP -two other adaptor molecules controlling the endocytic APP/BACE1 trafficking- also subserves the in vivo anti-amyloidogenic action of 12A12mAb. (**A**,**C**) Representative images of SDS-PAGE Western blotting analysis (*n* = eight animals per each group, four males and four females for each experimental condition) carried out on synaptosomal preparations of hippocampus and retina from animals of three experimental groups (littermate wild-type, vehicle-treated Tg2576, Tg2576+mAb) with antibodies for RIN3 and CD2AP (as indicated alongside the blots). Dashes on the right side indicate the molecular weight (kDa) of bands calculated from the migration of standard proteins. (**B**,**D**) Histograms show the semi-quantitative densitometry of the intensity signals of bands by normalization with β-actin level used as a loading control. *p* < 0.05 was accepted as statistically significant (one-way ANOVA followed by Bonferroni’s post hoc test for multiple comparisons among more than two groups * *p* < 0.05; ** *p* < 0.01; *** *p* < 0.0005).

Although its role in regulating the APP processing is currently under debate [68,91], CD2 Associated Protein (CD2AP) is another adaptor molecule involved in the APP trafficking from early endosomes to the lysosomal degradation pathway [92,93] and genetically linked with high risk of developing AD neuritic plaque pathology [94,95]. The depletion/loss of function of CD2AP causes the APP accumulation into early endosomes by retarding its delivery and degradation into lysosomes to enhance intraneuronal Aβ generation [68,96]. Additionally, the Rab INteractor 3 protein (RIN3)—a Guanine nucleotide Exchange Factor (GEF) identified as an additional risk factor underlying the genetic complexity of AD [65,96,97,98]—is reported to recruit CD2AP and BIN1 in a tripartite complex to Rab-5 GTPase-positive early endosomes [96,97,98,99] thus promoting the BACE1-dependent processing of APP in this subcellular compartment [91]. Therefore, in order to better characterize the impact of 12A12mAb immunization on the β-amyloidogenesis by immunoblotting with specific antibodies, we also compared the protein amount of RIN3 and CD2AP in synaptosomal preparations of hippocampus and retina from all three animals’ cohorts (Figure 2). As shown (Figure 2A,B), the intensity band of RIN3 showed a slight but statistically significant elevation in the hippocampus from Tg2576 AD mice when compared to age-matched wild-type littermates (* *p* < 0.05; Tg2576 versus wild-type), in agreement with the co-distribution of APP/BACE1 high signals we contextually detected in synaptic fractions (Figure 1A–D). These findings are also in line with the activation of amyloidogenic processing of APP (assessed as generation of βCTF fragment) in APP/PS1 mouse brains showing upregulation in RIN3 protein expression [96]. Conversely, an opposite pattern was unexpectedly detected in retinal samples since Tg2576 AD mice displayed a decrease in RIN3 immunoreactivity in comparison with wild-type control group (** *p* < 0.01; Tg2576 versus wild-type). Interestingly, 12A12mAb administration significantly normalized the changes in the RIN3 signals only in the retina (*** *p* < 0.0005; Tg2576+mAb versus Tg2576) since no noticeable modulation was contextually detected in the corresponding hippocampus from Tg2576 AD cohort when compared to its not-immunized counterpart (*p* > 0.9999; Tg2576+mAb versus Tg2576).

Despite its proposed role in regulating the intracellular endo-lysosomal transport of β-secretase and APP, no change in immunoreactivity levels among three experimental groups (*p* > 0.9999) was detected when the filters were probed with a specific antibody for CD2AP (Figure 2C,D), indicating that this protein is not involved in the anti-amyloidogenic effect of 12A12mAb immunization both in hippocampal and retinal synapses of Tg2576 AD mice.

**Figure 3:**
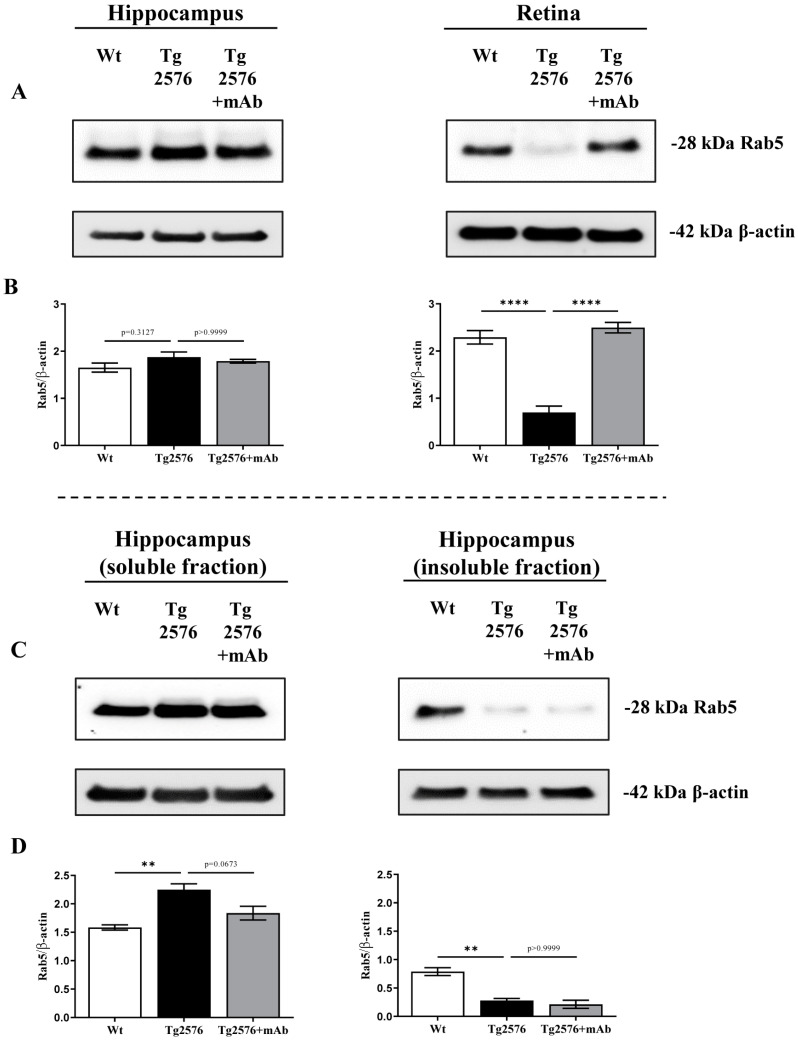
Deregulation of Rab5 GTPase is recovered only in the retina but not in the hippocampus of 12A12mAb-immunized Tg2576 AD mice. (**A**) Representative images of SDS-PAGE Western blotting analysis (*n* = six animals per each group, three males and three females for each experimental condition) carried out on synaptosomal preparations of hippocampus and retina from animals of three experimental groups (littermate wild-type, vehicle-treated Tg2576, Tg2576+mAb) with antibody for total Rab5 (as indicated alongside the blots). Dashes on the right side indicate the molecular weight (kDa) of bands calculated from the migration of standard proteins. (**B**) Histograms show the semi-quantitative densitometry of the intensity signals of bands by normalization with β-actin level used as a loading control. *p* < 0.05 was accepted as statistically significant (one-way ANOVA followed by Bonferroni’s post hoc test for multiple comparisons among more than two groups **** *p* < 0.0001). (**C**) Representative images of SDS-PAGE Western blotting analysis (*n* = six animals per each group, three males and three females for each experimental condition) carried out on Soluble and Insoluble synaptosomal fractions from the hippocampus of animals of three experimental groups (littermate wild-type, vehicle-treated Tg2576, Tg2576+mAb) with antibody for Rab5 (as indicated alongside the blots) to evaluate the protein repartitioning between cytosolic (inactive) and membrane-bound (active) forms. Dashes on the right side indicate the molecular weight (kDa) of bands calculated from the migration of standard proteins. (**D**) Histograms show the semi-quantitative densitometry of the intensity signals of bands by normalization with β-actin level used as a loading control [70]. *p* < 0.05 was accepted as statistically significant (one-way ANOVA followed by Bonferroni’s post hoc test for multiple comparisons among more than two groups ** *p* < 0.01).

Dysregulation of the endocytic pathway is a neuropathological hallmark of AD, with Rab5-positive early endosomes being the major site of APP processing by BACE1 [52,58,100]. However, the mechanism by which APP meets BACE1 is still largely unknown since both upregulation and downregulation of Rab5 GTPase activity are reported to increase Aβ production [52,58,101]. Additionally, RIN3 and/or BIN1 are reported to act through Rab5 in regulating endosomal trafficking and signaling as shown by the following in vitro and in vivo evidence: (i) In cell-based system BIN1.1 downregulates BACE1-mediated processing of APP into Rab5-positive early endosomes in a RIN3-dependent manner [102]; (ii) in primary cortical neurons upregulation of RIN3 expression promotes accumulation of APP-βCTFs and these effects are rescued by the expression of a dominant negative Rab5 (Rab5S34N) construct [91]; (iii) conditional knockout of BIN1 induces accumulation and enlargement of Rab5-positive early endosomes in mice [103]. In view of these data, our synaptosomal preparations of the hippocampus and retina were also checked for the Rab5 expression level by probing the filter with the specific antibody. As shown in Figure 3A,B and despite the obvious activation of β-amyloidogenesis (Supplementary Figure S1; Figure 1A,B), in the hippocampal specimens, no change in the total amount of Rab5 was apparently detected among three experimental groups (Tg2576 versus wild-type *p* = 0.3127; Tg2576+mAb versus Tg2576 *p* > 0.9999). On the contrary, in retinal samples, a dramatic decrease in Rab5 immunoreactivity was detected in Tg2576 AD mice when compared with wild-type control group (**** *p* < 0.0001; Tg2576 versus wild-type); following 12A12mAb administration and just as detected for BIN1 (Figure 1E,F) and RIN3 (Figure 2A,B), the steady state expression level of Rab5 was significantly recovered in Tg2576 AD cohort when compared to its not-immunized counterpart (**** *p* < 0.0001; Tg2576+mAb versus Tg2576). After that, and because the rate of Rab5 distribution between cytosol and membrane compartments provides a reliable indication of its activation state [104], we better deepened the role of this endocytic protein by measuring its reciprocal separation into soluble (inactivated, GDP-bound)/insoluble (activated, GTP-bound) fractions from hippocampal protein extracts. As shown in Figure 3C,D, a substantial decrease in the active form of Rab5 was found in Tg2576 AD mice when compared with wild-type control group (** *p* < 0.01; Tg2576 versus wild-type), whereas no statistically significant effect was detected in the immunized cohort following 12A12mAb treatment (*p* > 0.9999; Tg2576+mAb versus Tg2576).

Taken together, these results indicate that 12A12mAb-mediated neutralization of the neurotoxic NH_2_htau exerts in vivo, in hippocampus and retina from Tg2576 AD mice, an anti-amyloidogenic action involving, respectively, the upregulation in the synaptic expression of neuron-specific BIN1 alone or together with RIN3, two key endocytic adaptors governing in opposite ways the dynamic convergence of APP and BACE1 into Rab5-positive endosome and then the Aβ generation.

### 2.2. Energetic Alterations of Glucose Utilization That Are Strictly Linked with the Aβ Generation Are Recovered by 12A12mAb Treatment Both in Retina and Hippocampus in Concomitance with Its Local Anti-Amyloidogenic Action

Both brain and eye preferentially use glucose as their main energy substrate [105,106] and a decline in cerebro-retinal utilization of glucose has been described to occur in AD transgenic animal models and affected patients as well, even before Aβ plaque deposition and the manifestation of clinical symptoms [107,108,109,110,111,112]. During physiological aging, L-lactate production increases due to reduced oxidative phosphorylation from mitochondrial dysfunction [113] and, more importantly, the N-terminal domain of tau—which is antagonized in vivo by the 12A12mAb—binds to mitochondria and impairs the ATP production by oxidative phosphorylation (OXPHOS) leading to increased ROS production, as we reported in our previously studies [45]. Additionally, under pathological conditions, a tight positive correlation between interstitial levels of L-lactate—the main metabolite of glycolysis—and Aβ load have also been reported in brains from preclinical AD mouse models [114,115,116]. In line with this, cortical and hippocampal L-lactate amounts increase with disease progression in the APP/PS1 transgenic AD mouse model of AD [117] and accumulation of secreted L-lactate takes place in the brain tissues from AD subjects and in the conditioned media from induced neurons (iN) from patient-derived fibroblasts [118]. L-lactate levels are also elevated in the brains of amnestic Mild Cognitive Impairment (MCI) patients [119] and in the CSF of AD patients [120,121,122]. Based on these findings, regulation of glycolytic production of L-lactate was considered a likely contributing factor to the anti-amyloidogenic action exerted in vivo by 12A12mAb [15,44].

**Figure 4:**
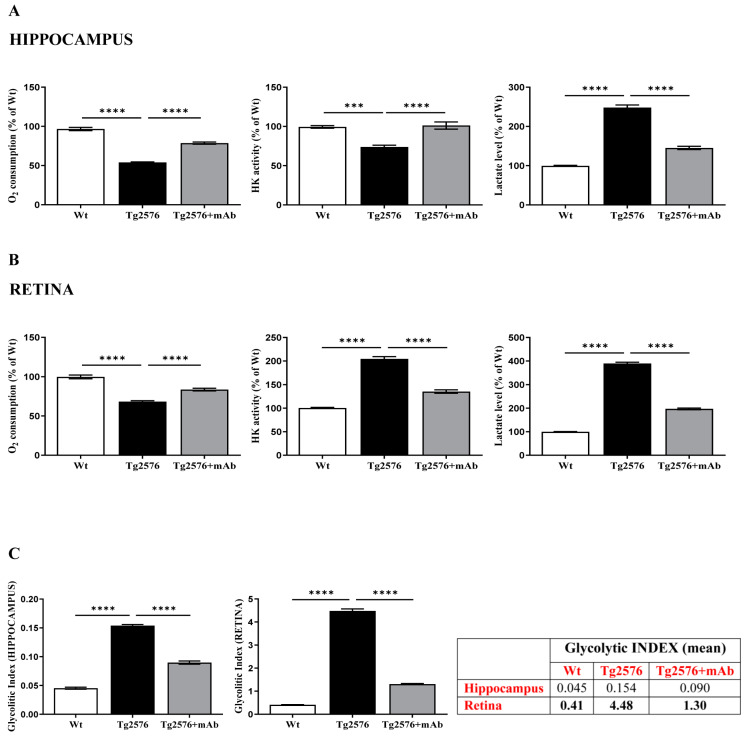
Hippocampal and retinal alterations of energetic metabolism of glucose are mitigated by 12A12mAb treatment in concomitance with its local anti-amyloidogenic action. (**A**,**B**) Hippocampal and retinal homogenates from animals of three experimental groups (littermate wild-type, vehicle-treated Tg2576, Tg2576+mAb) were assessed for glucose metabolism (*n* = six animals per each group, three males and three females for each experimental condition). On the left: O_2_ consumption. In the middle: determination of HexoKinase (HK) activity. On the right: assessment of Lactate levels in the hippocampus (**A**) and retina (**B**), respectively. (**C**) Glycolitic index in the hippocampus (left) and in the retina (right). *p* < 0.05 was accepted as statistically significant (one-way ANOVA followed by Bonferroni’s post hoc test for multiple comparisons among more than two groups *** *p* < 0.0005; **** *p* < 0.0001).

Thus, we evaluated the utilization of glucose along with oxygen consumption and L-lactate production in parallel, both in the hippocampus and retina, from three experimental groups (wild-type, Tg2576 and Tg2576+mAb) (Figure 4). Functional homogenates—which contain intact and coupled mitochondria [123]—were carried out because they: (i) provide a more ‘physiological’ environment for the investigation of GLU flux through the glycolytic pathway in situ; (ii) are suitable for monitoring as both the glycolytic and mitochondrial machineries contextually are closely interlinked. First, the ability of our preparations to oxidize glucose was investigated by monitoring the oxygen uptake after the incubation of homogenates with glucose. In a first preliminary set of experiments, the addition of Glucose (GLU, 5 mM) to mouse tissue homogenates results in oxygen consumption as a consequence of a multistep process consisting of GLU entry into the glycolytic pathway, pyruvate production and uptake by mitochondria where citric cycle and oxidative phosphorylation operate with oxygen consumption. Therefore, the addition of cyanide—a classical and strong inhibitor of cytochrome oxidase (COX) activity—totally prevented oxygen uptake, consistent with the enzyme subcellular localization along this metabolic pathway. Finally, the incubation of homogenates with 3-bromopyruvate—a fast and powerful inhibitor of Hexokinase (HK), which is a rate-limiting enzyme catalyzing the first step in the process of GLU conversion to Glucose-6-Phosphate (G6P) and its oxidation along the glycolytic route—caused a decrease in oxygen uptake [123].

After checking the integrity/quality of our homogenate preparations, we measured the O_2_ consumption expressed in atoms O/min × mg protein and calculated as % of control. As shown in Figure 4A,B (on the left), the oxidation rate of GLU in homogenates from both hippocampus and retina of Tg2576 mice drastically decreased (about 45% and 30% with the oxygen consumption rate values equal to 32 and 24 atoms O/min × mg, respectively) compared to that of corresponding wild-type controls (hippocampus **** *p* < 0.0001; retina **** *p* < 0.0001; Tg2576 versus wild-type). Systemic administration of 12A12mAb to Tg2576 mice significantly increased the oxygen uptake values close to wild-type ones (hippocampus **** *p* < 0.0001; retina **** *p* < 0.0001; Tg2576+mAb versus Tg2576).

Then, we carried out direct determination of HK trapping GLU into G6P along the glycolytic cascade (Figure 4A,B in the middle) and we found out that its activity was lower in the hippocampus (25% decrease) and higher in the retina (100% increase) from Tg2576 mice when compared with their controls (hippocampus *** *p* < 0.0005; retina **** *p* < 0.0001; Tg2576 versus wild-type). Nevertheless, and more importantly, an opposite trend was detected in vivo following the 12A12mAb administration in Tg2576 AD mice: an increase in the glycolytic enzyme activities (approximately 30%) was clearly found in the hippocampus, whereas an inverse drop was detected in the retina (approximately of 65%) (hippocampus **** *p* < 0.0001; retina **** *p* < 0.0001; Tg2576+mAb versus Tg2576).

Finally, the level of L-lactate (Lactate) was measured in our preparations (Figure 4A,B, on the right). As expected, the amount of L-lactate resulted elevated both in hippocampal and, even more, retinal homogenates from Tg2576 AD mice in comparison with their wild-type littermates (about 2.5 and 4 times in hippocampus and retina, respectively; hippocampus **** *p* < 0.0001; retina **** *p* < 0.0001; Tg2576 versus wild-type).

These findings extend previous findings [25,106], confirming that, under physiopathological conditions, glucose metabolism is quite different along the neurosensorial circuit (brain-eye). In particular, in the retina of Tg2576 mice, the increase in L-lactate occurs concomitantly with an upregulation of HK—a key enzyme that internalizes and metabolizes glucose—resembling thus a “reinforced Warburg effect” (i.e., the use of glucose outside oxidative phosphorylation beyond that predicted by oxygen consumption) since the local glucose metabolism is already upregulated by itself in the photoreceptor cells [106,124,125,126]. On the other hand, the increase in the L-lactate level in their diseased hippocampus hints at the local occurrence of anaerobic glycolysis due rather to the impairment of mitochondrial oxidative phosphorylation, which is known to be the preferential bioenergetic route into post-mitotic neurons [123,127]. It is noteworthy that 12A12mAb immunization significantly decreased the level of L-lactate both in the hippocampus and retina from Tg2576 AD mice, indicating that in vivo treatment with this tau antibody strongly normalized the GLU metabolism along the glycolytic route approximately to control values (hippocampus **** *p* < 0.0001; retina **** *p* < 0.0001; Tg2576+mAb versus Tg2576).

Having assessed the decrease in oxygen consumption along with the increase in GLU utilization and the accumulation of L-lactate (L), we finally calculated the glycolytic index (GI) in both tissues (Figure 4C). The GI value—which describes the degree to which a cell uses glycolysis to meet its total ATP demand—gives insights into the extent of glycolysis. As shown, in the retina from Tg2576 AD mice, the GI strongly increased (from 0.41 to 4.48) in comparison with wild-type counterparts (**** *p* < 0.0001; Tg2576 versus wild-type), whereas this value diminished by 34% (up to 1.30) following 12A12mAb delivery (**** *p* < 0.0001; Tg2576+mAb versus Tg2576). In the hippocampus, a similar trend was detected but at an extent much lower (0.045 wild-type; 0.154 Tg2576; 0.090 Tg2576+mAb) (**** *p* < 0.0001; Tg2576 versus wild-type, Tg2576+mAb versus Tg2576). Indeed, the percentage difference of GI value in Tg2576 hippocampus with respect to the corresponding retina indicates that in the brain, the glucose may be metabolized more efficiently in terms of ATP generation, with a greater reliance on mitochondria-based oxidative metabolism.

Collectively, our results confirm and extend previous investigations [25,125] by showing that: (i) retinal ganglion cells show different energetic profiles and metabolic re-programming in comparison with pyramidal hippocampal neurons (cytosolic glycolysis versus mitochondrial oxidative phosphorylation, respectively), both under physiological and pathological conditions; (ii) the in vivo anti-amyloidogenic action exerted by 12A12mAb is associated with normalization of the lactate production and, then, the Glycolitic Index (GI) both in brain and retina.

## 3. Discussion

A growing body of evidence indicates that BACE1 and APP converge in early, Rab5-positive endosomes of neurons where the BACE1-mediated amyloidogenic cleavage of APP mainly occurs [50,100,128,129,130,131,132,133,134,135,136,137,138,139]. Thus, endosomal trafficking dysfunction usually leads to the endosomal accumulation of BACE1 or APP and enhances the Aβ generation [68,140]. Consistent with these findings, endosomal impairment is increasingly viewed as a key cellular phenotype contributing to AD pathogenesis [60,141,142]. In this context, a relevant observation of the present work is that 12A12mAb exerts an anti-amyloidogenic effect in vivo [15,44] both in the CNS and neurosensory retina (Supplementary Figure S1) along a pathway(s) involving several AD-associated risk variants coding for BIN1 and RIN3 (Figure 1 and Figure 2), two crucial protein regulators of clathrin-mediated endocytic trafficking [98,143]. On the one hand, immunization of symptomatic Tg2576 AD mice normalizes in the retina even up to the control values the steady state expression levels of BIN1 and RIN3, which regulate in a reciprocal way (inhibition and stimulation, respectively) the APP/BACE1 sorting convergence into Rab5-positive early endosomes (i.e., initial APP cleavage by BACE1 leading to Aβ generation) [67,68,91,144]. On the other hand, in vivo treatment with our cleavage-specific tau antibody successfully recovers in the hippocampus the decrease in protein amount of BIN1 in the absence of any effect on concomitant deregulation in RIN3 and active Rab5 expression. Although the pathophysiological significance of this difference between the retina and hippocampus of Tg2576 AD mice in response to 12A12mAb administration has yet to be elucidated, it is noteworthy that the same endocytic pathway that crucially regulates the BACE1-triggered APP degradation at synapses is engaged by 12A12mAb in both tissues to limit the side-by-side Aβ production. In line with our in vivo results is the in vitro observation that neuronal BIN1 negatively regulates the endocytic transport of BACE1 to recycling endosomes so that, when it is downregulated, BACE1 aberrantly accumulates into Rab5-positive early endosomes, causing an augmented Aβ production [67,68,145]. Both increase and decrease in BIN1 expression levels have been described in the post-mortem AD brains and animal models [69], although experimental differences in methodology used, animal strains and human brain tissues analyzed should be taken into account for this discrepancy. However, even though a more recent study indicates that suppression of BIN1 function does not regulate the Aβ generation in 5xFAD mice [146], the level of neuronal BIN1 protein turns out to be significantly decreased in brains from AD-affected cases with evident β-amyloidogenesis [80,147,148,149]. Interestingly, BIN1 directly binds to the proline-rich domain of tau via its SH3 domain [147,150,151] and interferes with its neurotoxicity, both in cellular and mouse models [69,152,153]. Above all, the N-terminus projection domain of tau—which is specifically targeted/antagonized in vivo by 12A12mAb [15,44]—is capable of binding to synaptic vesicles and to several proteins involved in clathrin-mediated endocytosis and synaptic trafficking, including BIN1 itself [70,71,72], both directly [154,155] and/or indirectly [156,157]. BIN1, which has a specific neuronal expression and mainly a presynaptic localization just as the 12A12mAb-targeted NH_2_htau [158], participates in the neuron-to-neuron, prion-like propagation of tau strains by modulating its endocytic flux, further standing up for their reciprocal interaction at synaptic compartment [71]. In addition, and consistent with our biochemical results, recent studies have also shown that RIN3 binds the neuronal isoform of BIN1 and that its upregulation enhances the production of neuronal toxic APP-βCTFs and tau (hyper)phosphorylation [96,147]. Accordingly, BIN1 reduces the β-secretase-mediated processing of APP exclusively in the neurons in a RIN3-dependent manner [102], providing interesting mechanistic insights on how these two AD-associated endocytic molecules controlling the Aβ production can mediate in vivo the anti-amyloidogenic effect of 12A12mAb [159]. Concerning the involvement of Rab5 cooperating with BIN1 and RIN3 in the regulation of intracellular vesicular trafficking, both its activation and inactivation have been found to correlate with a stimulated amyloidogenic pathway and augmented Aβ production in AD pathogenesis. On one side, an upregulation of Rab5 along with enlargement of early endosomes are detected in different vulnerable brain regions of affected subjects [48,160,161] and hyperstimulation of Rab5 accelerates the generation of Aβ in vitro [135]. On the other side, extracellular Aβ levels are increased when the expression of Rab5 is knocked down by siRNA leading to prolonged APP-BACE1 interaction in neurons [101] and the expression of dominant negative Rab5 mutant rescues deficits of neuronal axonal transport caused by overproduction of APP and β-CTF in Drosophila [58]. The finding that the marked downregulation of Rab5 expression level from Tg2576 is significantly recovered by 12A12mAb administration in the retina in concomitance with a local reduction in Aβ production (Figure 3) is consistent with the latter findings. On the contrary, the evidence that the diminution of GTP-bound membrane active form of Rab5 we contextually detected in the hippocampus does not respond to antibody treatment in immunized AD mice hints at the involvement of other adaptors proteins of the endocytic pathway controlling the APP/BACE1 trafficking (i.e., SorLA) into neurons [162,163] in 12A12-mediated beneficial effects on cerebral Aβ accumulation.

Another interesting aspect of this study is that the anti-amyloidogenic action exerted in Tg2576 by 12A12mAb involves, at least in part, the local mitigation of the energetic dyshomeostasis, both in the hippocampus and retina. In particular, we found out that 12A12mAb immunization reduces in vivo the Aβ accumulation (Supplementary Figure S1) [15,16,17,18,19,20,21,22,23,24,25,26,27,28,29,30,31,32,33,34,35,36,37,38,39,40,41,42,43,44] by normalizing to basal levels the L-lactate (Figure 4), which accumulates in neurons along the glycolytic route in response to mitochondrial inhibition [164] and promotes the deposition of Aβ protein [165,166]. Although it is difficult to establish whether brain hypometabolic status is a consequence or cause of AD pathology, a consistent and progressive reduction in brain bioenergetics (glucose hypometabolism and mitochondrial dysfunction) is a well-established [167], robust hallmark of AD staging since its extent and topography correlate with the Aβ deposition [168,169] and with the severity of clinical symptoms of dementia [170,171,172]. Increased aerobic glycolysis (i.e., glycolytic activity, which is not matched by oxidative phosphorylation, known as the “Warburg effect”) is visible in specific AD brain regions, which are more susceptible to Aβ accumulation [123,173]. Consistently, elevated levels of L-lactate have been detected in two APP-overexpression transgenic rat models of AD upon exposure to isotope-radiolabeled glucose [107,174], in agreement with similar results in AD patients [175,176]. Additionally, energetic metabolism stimulates per se the BACE1 protein amount and activity towards the amyloidogenic processing of APP both in vitro [177,178] and in vivo [85,179,180], proving that its global impairment performs a key part in the neuro-sensorial accumulation of Aβ and its amyloidogenic derivates [169,181,182,183]. More importantly, we have reported that an in vivo convergence of tau and Aβ pathologies take place at AD mitochondria since the NH_2_htau synergically cooperates with Aβ species in inhibiting the oxidative phosphorylation synthesis of ATP, leading to the energy crisis and, eventually, neuronal deterioration [45]. In this regard, an intriguing recent study shows that a Warburg-like aerobic glycolytic transformation occurs in the brains of AD patients and that this metabolic transformation is not merely an adaptation to mitochondrial defects but is specifically activated to unfold an apoptotic program. The authors report that pharmacological modulation of the isoform 2 of Pyruvate kinase (PKM2)—one of the key enzymes of glycolysis acting on Phos-phoEnolPyruvate (PEP) to form pyruvate that is then converted in L-lactate by Lactate DeHydrogenase (LDH)—prevents neuronal death by normalizing the neuronal secretion of L-lactate to basal levels [118]. From a translational point of view, these results seem to fit well with our metabolic data hinting at tau-dependent energetic changes of glucose utilization along the glycolytic (i.e., lactate production) and mitochondrial pathways as key mediators of the neuroprotective and anti-amyloidogenic effect of 12A12mAb immunization in Tg2576 AD animal model. Interestingly, RNA-seq transcriptomic profiles of wild-type, Tg2576 and Tg2576+12A12mAb mice (Gene Expression Omnibus accession number GSE223593, manuscript in preparation) show that the downregulation of mitochondrial energetic pathway both in hippocampus and retina is significantly (FDR *p*-value < 0.001) reverted by treatment with 12A12mAb, further supporting its in vivo anti-amyloidogenic action.

**Figure 5:**
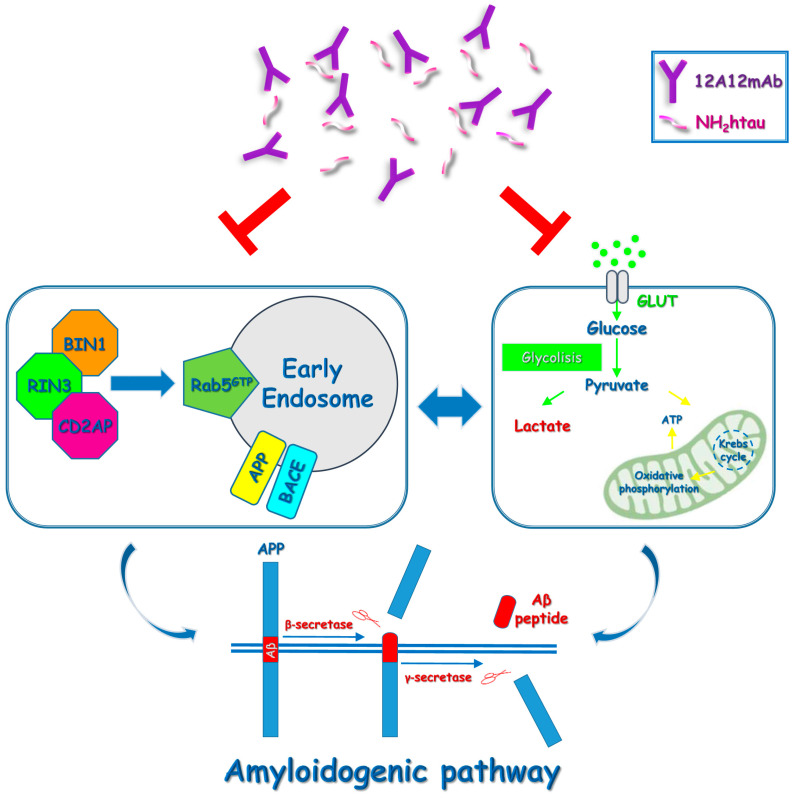
Proposed model of 12A12mAb-mediated signaling pathways taking part both in hippocampus and retina from symptomatic Tg2576 AD mouse model. In vivo targeting of the NH_2_htau by 12A12mAb exerts an indirect anti-amyloidogenic action by normalizing the deregulation of: (a) the RAB5-mediated endocytosis and (b) the mitochondrial/glycolytic energetic pathways, which cooperate in triggering the neurosensorial accumulation of Aβ and its amyloidogenic derivates.

In view of these findings concerning the effect of tau-directed immunization on the APP/Aβ metabolism, it is tempting to speculate (Figure 5) that the soluble pathological tau species, including the neurotoxic 12A12mAb-targeted NH_2_htau, could disrupt the vesicular trafficking of APP along its amyloidogenic processing towards the Aβ generation both directly (via endocytic adaptors) and/or indirectly (via mitochondrial impairment and increased L-lactate production).

Finally, there are several aspects of our study that deserve additional comments. First, our molecular and metabolic results, due to technological issues (whole hippocampus and retina), are limited by protein abundance and energetic index averaging among the multiple tissue populations included in both specimens, hampering thus the exploration of cell-type specific molecular alterations. Second, although changes in the BIN1 and RIN3 regulators perturb the intracellular sorting of APP and BACE1 and promote the amyloidogenic processing [67,68,96,184,185,186], it is unclear whether 12A12mAb regulates endosomal dysfunction with Aβ accumulation directly or if other, still unidentified, factors to perform a role. Finally, we cannot rule out that 12A12mAb may concomitantly increase the Aβ clearance or degradation and, thus, further studies aimed to examine the in vivo Aβ turnover in Tg2576 are needed to address this important point.

## 4. Materials and Methods

### 4.1. Animals and Ethical Approval

All animal experiments complied with the ARRIVE guidelines and were carried out in accordance with the ethical guidelines of the European Council Directive (2010/63/EU); experimental approval was obtained from the Italian Ministry of Health (Authorization n. 524/2017 PR; Authorization n. 1038-2020-PR). This study was carried out according to the principles of the 3Rs (Replacement, Reduction and Refinement).

Heterozygous Tg2576 mice of both genders (Tg-AD), expressing the human Amyloid Precursor Protein (APP) with the Swedish mutation KM670/671NL [187], which causes an increase in Aβ production [188] and their wild-type (Wt) littermates were used at 6 months of age (*n* = 8–10 per group) in the immunization regimen. Genotyping was carried out to confirm the presence of human mutant APP DNA sequence by PCR.

### 4.2. Immunization Scheme

The N-terminal tau 12A12 monoclonal antibody (26–36 aa) was produced and purified from hybridoma supernatants according to standard procedures, as previously described in [15,44].

The mice were randomized into (1) wild-type mice treated with saline vehicle; (2) age-matched Tg2576 mice treated with saline vehicle and (3) age-matched Tg2576 mice treated with 12A12mAb (30 μg/dose). Animals were infused over 14 days with two weekly injections administered on two alternate days to the lateral vein of the tail. The dose and route of immunization were based on previously published studies by our and other independent research groups using Tg2576 as AD transgenic mouse model [15,44,189].

Notably, this immunization regimen was previously demonstrated to successfully deliver in vivo a sufficient amount of biologically active (antigen-competent) tau antibody to promote the clearance of the deleterious NH_2_htau peptide accumulating into animals’ hippocampus and retina and to significantly alleviate their behavioral, biochemical (accumulation of APP/Aβ and tau hyperphosphorylation), electrophysiological and morphological disease-associated neurosensorial signs. No inflammation was detected—both in the brain and eye—following 14 days of antibody treatment [15,44].

### 4.3. Tissue Collection, Harvesting and Preparation

For biochemical analysis:

Two days after the last injection of 12A12mAb [15,44], animals from three experimental groups (wild-type, vehicle-treated Tg-AD, Tg-AD+mAb) were sacrificed by cervical dislocation, perfused transcardially with ice-cold phosphate-buffered saline (PBS), brains and eyes were collected, hippocampi and retinas were dissected, immediately frozen on dry-ice and then stored at −80 °C until use.

Crude synaptosomal preparations were obtained from mice of three experimental groups (wild-type, vehicle-treated Tg-AD, Tg-AD+mAb), as reported [190].

Preparation of soluble and insoluble fractions for detection of the level of membrane-bound (GTP-active loaded form) relative to cytosolic (GDP-bound inactive form) Rab5 was carried out as previously reported [191]. In detail, synaptosomal lysates were separated by high-speed centrifugation (100,000× *g*) for 1 h at 4 °C for recovery of membrane proteins as for water-soluble proteins. Pellet (insoluble) was resuspended to an equal volume of supernatant (soluble) in RIPA buffer (50 mM Tris–HCl, pH 8, 150 mM NaCl, 1% Triton, 2 mM EDTA, 0.1% SDS plus proteases inhibitor cocktail (P8340, Sigma Aldrich, St. Louis, MO, USA) and phosphatase inhibitor cocktail (P5726/P2850, Sigma-Aldrich,). β-actin was used as a loading control [192].

For histopathological analysis, animals were intracardially perfused with ice-cold phosphate-buffered-saline (PBS) 0.1 mol/L pH7.4 using a 30 mL syringe to remove blood contamination and then with 4% paraformaldehyde (PFA) solution in PBS. The hippocampus and eye were isolated, cleaned with PBS with utmost caution not to inflict damage and dipped in tubes containing 10% neutral buffered formalin solution (F0048, Diapath, BG, Italy) for the purpose of post-fixation. Tubes were left over at room temperature until to be used.

### 4.4. Western Blot Analysis and Semi-Quantitative Densitometry

Equal amounts of protein extracts (80–150 μg) were size-fractionated by SDS-PAGE Bis-Tris gel 4–12% (Bolt, ThermoFisher Scientific, Waltham, MA, USA) according to [15,44]. β-actin was used as an internal control of protein loading and semi-quantitative densitometric analysis was carried out by using Image J 1.4 (http://imagej.nih.gov/ij/ accessed on 1 October 2012). For quantification, we measured the band intensity by using a signal in the linear range.

SDS-PAGE was carried out on 10–20% Tricine gels (Novex, Invitrogen) with 0.1 μm nitrocellulose membrane for the detection of 4 kDa Aβ monomer(s) and its products, as previously described [193].

The following antibodies were used:

Anti-synaptophysin antibody (D-4) mouse sc-17750 Santa Cruz; anti-syntaxin 1 mouse S1172 Sigma-Aldrich; anti-SNAP25 antibody (clone SMI 81) mouse 836301 BioLegend; anti-α synuclein antibody (clone 42) mouse 610786 BD Transduction Laboratories; GAPDH antibody (6C5) mouse sc-32233, Santa Cruz; NeuN antibody (clone A60) mouse MAB377, Millipore; c-Fos antibody (9F6) rabbit 2250S, Cell Signaling; tau antibody (BT2) mouse MN1010 ThermoFisher Scientific; anti-Amyloid Precursor Protein 22C11 (66–81 aa of N-terminus) mouse APP-MAB348 Chemicon; BACE-1 (61-3E7) mouse sc-33711 Santa Cruz; BIN1 (amphiphysin II, 99D) mouse sc-13575 Santa Cruz; anti-RIN3 rabbit 12709-1-AP Proteintech; CD2AP rabbit 51046-1-AP Proteintech; anti-Aβ/APP protein 6E10 (4–9 aa) mouse MAB1560 Chemicon; anti-Aβ amyloid specific (D54D2) rabbit 8243 cell Signaling; anti-Rab 5 (D-11) mouse sc-46692 Santa Cruz; anti-β-actin antibody mouse S3062 Sigma-Aldrich; anti-mouse IgG (whole molecule)-Peroxidase antibody A4416 Sigma-Aldrich (St. Louis, MO, USA) and anti-rabbit IgG (whole molecule)-Peroxidase antibody A9169 Sigma-Aldrich (St. Louis, MO, USA).

### 4.5. Hematoxylin and Eosin (H/E) Staining

Hematoxylin and eosin staining was carried out as previously reported [15] with some modifications. In detail, tissues were shifted to melted paraffin wax and solidified. Several sections of the tissues of 5 μm thickness were manually trimmed using a microtome (SM200MR, Leica Microtome, Milan, Italy) with steel disposable blades (FEATHER S35, PFM Medical, Carlsbad, CA, USA). The tissue slices were subsequently dewaxed, followed by dehydration with increased gradient concentrations of an aqueous-alcohol solution. The slices were stained with hematoxylin (Gill 3) and eosin dye (05-06015/L, Bio-Optica, Milan, Italy) by automatic stainer (Leica ST5020), placed on glass slides on quick mounting medium for the histological technique (Eukitt, ORSAtec, Bobingen, Germany) and observed under a light microscope.

### 4.6. Glucose Metabolism Analysis

#### 4.6.1. Tissue Homogenate Preparation

Both brains (hippocampi) and retinas from three experimental groups were stored at −80 °C until assayed. All assays were performed on freshly homogenized tissue samples containing mitochondria. The PBI-Shredder—an auxiliary high-resolution respirometry (HRR) Tool—was used to prepare homogenate of frozen tissue specimens in 0.2 M phosphate buffer (pH 8.0) according to [15], with high reproducibility of the mitochondrial function evaluated with HRR by means of Oxygraph-2 k OROBOROS^®^ (Innsbruck, Austria). Then, appropriate checks were made to ensure the complete rupture of the plasma membrane and, at the same time, the integrity of the mitochondrial ones. Homogenate protein content was determined as in [194].

#### 4.6.2. Enzymatic Activity Measurements

HexoKinase (HK) activity was assayed spectrophotometrically at 340 nm (e334 nm = 6.22 mM^−1^ cm^−1^) using a Jasco double-beam/double-wavelength spectrophotometer UV-550 under Vmax conditions according to procedures briefly described in [195,196].

#### 4.6.3. L-Lactate Production Measurements

Determination of L-lactate content was performed in homogenates of the hippocampus and retina by the enzymatic photometric method of Brandt [195]. L-Lactate, the metabolite of anaerobic glycolysis, can be measured indirectly by using the activity of Lactate Dehydrogenase (LDH). LDH converts/oxidizes lactate to pyruvate by using oxidized Nicotinamide Adenine Dinucleotide (NAD^+^) as co-factor (lactate + NAD^+^ -> pyruvate + NADH/H^+^). Thus, the difference in absorbance at 340nm can be used to calculate lactate level by using the extinction coefficient for reduced Nicotinamide Adenine Dinucleotide (NADH) equal to 6.22.

#### 4.6.4. Determination of the Glycolytic Index

The tissue glycolytic activity was evaluated, according to [195,196], by measuring the glycolytic index (GI), which was calculated by the formula: GI = (L×G)/O, where L is the L-LAC production, G is GLU utilization rate, i.e., the HK activity in this case, and O is the oxygen consumption rate. All measurements were conducted under roughly similar conditions.

### 4.7. Data Management and Statistical Analysis

Values were expressed as means ± standard error of the mean (S.E.M.) Statistically significant differences were calculated by one-way analysis of variance (ANOVA) followed by Bonferroni’s post hoc test for multiple comparisons among more than two groups. *p* < 0.05 was accepted as statistically significant (* *p* < 0.05; ** *p* < 0.01; *** *p* < 0.0005; **** *p* < 0.0001). The sample size was estimated on the basis of our previously published experiments [15,44] reporting changes in Tg2576 and age-matched wild-type littermate mice after 12A12mAb immunization. An “a priori” estimation to compute the required sample size by a given α power and effect size was carried out by G*Power statistical power analysis (version 3.1.9.4). All statistical analyses were performed using GraphPad Prism 8 software.

## 5. Conclusions

Our results indicate that the local anti-amyloidogenic action exerted in vivo, both in the hippocampus and retina from symptomatic Tg2576 AD mouse model, by the cleavage-specific tau 12A12mAb entails a concomitant modulation in: (i) two key adaptors of the clathrin-mediated endocytic pathway (BIN1, RIN3) which control the BACE1-triggered APP maturation along the β/γ-mediated amyloidogenic route; (ii) the metabolic utilization of glucose along the glycolytic route (i.e., L-lactate production) and the mitochondrial oxidative phosphorylation pathway.

In summary, the present study aimed at understanding the molecular mechanisms underlying the beneficial action of 12A12mAb will not only move forward its preclinical development to a candidate for the treatment of patients affected by AD but also will improve our knowledge of the tau pathobiology in vivo in the context of human tauopathies.

## Data Availability

All the data used and/or analyzed for the current study is contained in the article. All other datasets are available from the corresponding author upon reasonable request.

## Supplementary Materials

The following supporting information can be downloaded at: https://www.mdpi.com/article/10.3390/ijms24119683/s1. References [15,197,198,199,200,201] are cited in the supplementary materials.

## Abbreviations

Alzheimer’s Disease (AD); monoclonal Antibody (mAb); IntraVenous (i.v.); N-terminal 20–22 kDa tau fragment (s) (NH_2_htau); Amyloid-β peptides (Aβ); Amyoid Precursor Protein (APP); Amyloid Precursor Protein KM670/671NL Swedish mutation (APPSwe); beta-site Amyloid Precursor protein Cleaving Enzyme 1 (BACE1); Transgenic (Tg); Wild-type (Wt); Central Nervous System (CNS); CerebroSpinal Fluid (CSF); Mild Cognitive Impairment (MCI); Bridging INtegrator 1 (BIN1); CD2 Associated Protein (CD2AP); Rab INteractor 3 protein (RIN3); Guanine nucleotide Exchange Factor (GEF); GuanosineTriphosphate nucleotide (GTP); Guanosine Diphosphate nucleotide (GDP); Ras-related protein 5 (Rab5); Sodium Dodecyl Sulfate-PolyAcrylamide Gel Electrophoresis (SDS-PAGE); Glucose (GLU); Cytochrome OXidase (COX); Hexokinase (HK); Glucose-6-Phosphate (G6P); Glycolitic Index (GI); Lactate DeHydrogenase (LDH); oxidized and reduced Nicotinamide Adenine Dinucleotide (NAD+ and NADH, respectively); Hematoxylin and Eosin (H/E).
