# Supplementary material for: The Cleavage-Specific Tau 12A12mAb Exerts an Anti-Amyloidogenic Action by Modulating the Endocytic and Bioenergetic Pathways in Alzheimer’s Disease Mouse Model
Source: Int J Mol Sci. 2023 Jun 2;24(11):9683. doi: 10.3390/ijms24119683 (PMC10253571; doi:10.3390/ijms24119683)
Supplement: Supplementary file 1 [file ijms-24-09683-s001.zip › ijms-2280072-supplementary.pdf]

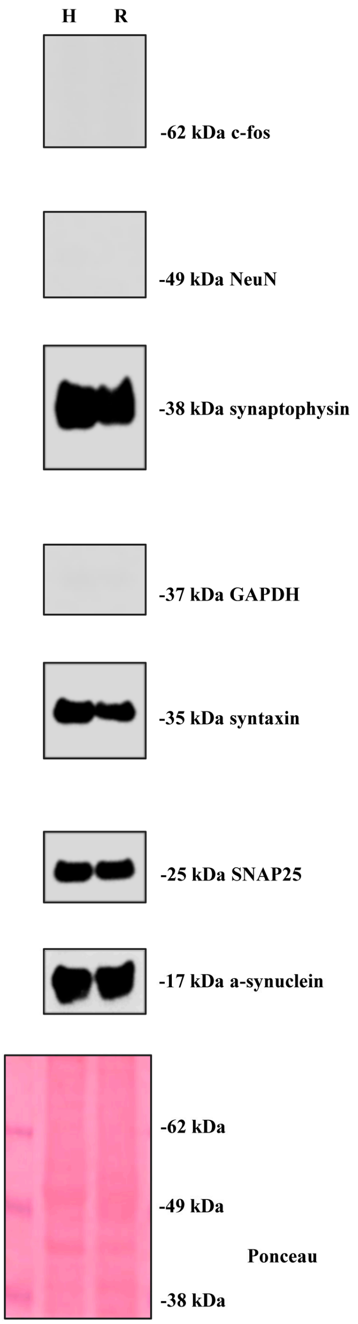

Supplementary Figure S1. Quality check of the purity of isolated crude synaptosomal fractions.

SDS-PAGE Western blotting analysis carried out on synaptosomal preparations of hippocampus (H) and retina (R) from wild-type animals (n=6) with antibodies for synaptic (synaptophysin, syntaxin, SNAP25, -synuclein), nuclear (c-fos, NeuN) and cytosolic (GAPDH) markers. Ponceau S staining was used for total protein normalization. Dashes on the right side indicate the molecular weight (kDa) of bands calculated from migration of standard proteins. Notice that synaptosomal preparations are free of cytosolic and nuclear proteins.

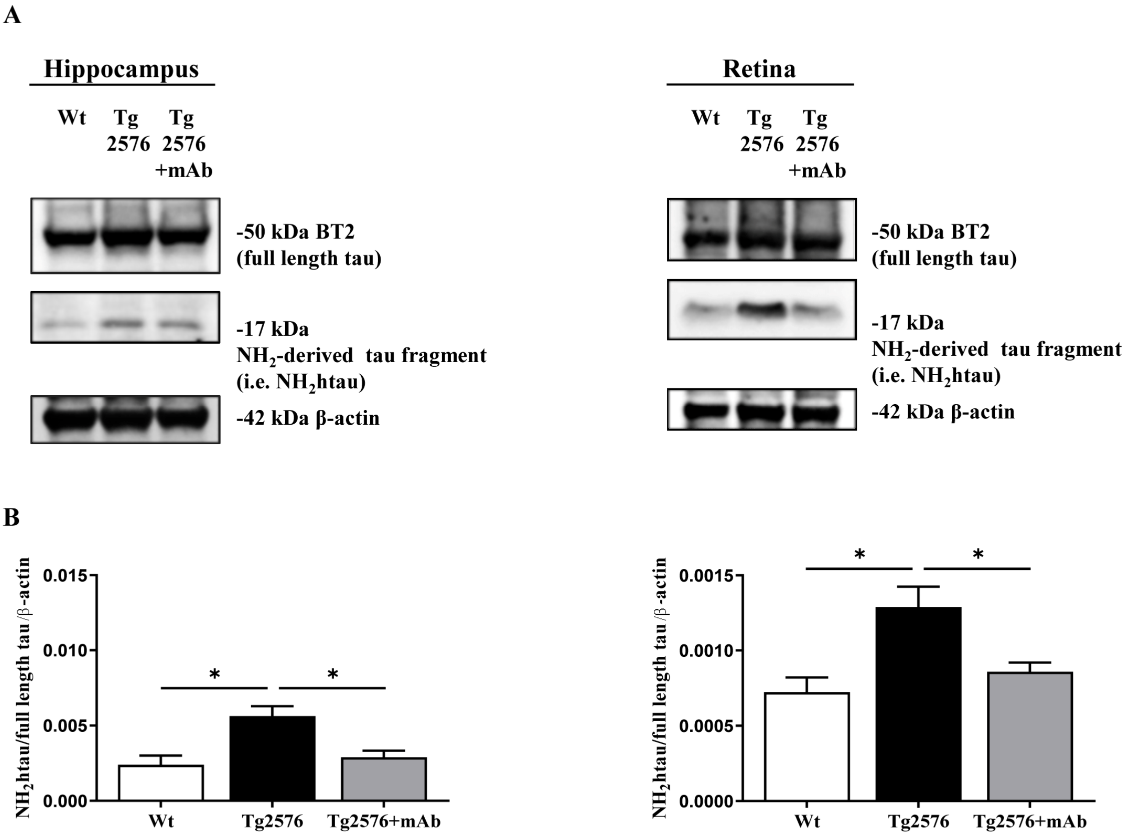

Supplementary Figure S2. Abnormal N-terminal cleavage of tau both in hippocampus and retina from Tg2576 AD mice is successfully antagonized by *in vivo* immunization with 12A12mAb.

(**A**) Representative images of SDS-PAGE Western blotting analysis (n = 6 animals per each group, 3 males and 3 females for each experimental condition) carried out on synaptosomal preparations of hippocampus and retina from animals of three experimental groups (littermate wild-type, naive/vehicle-treated Tg2576, Tg2576+mAb) with BT2 (194-198aa), a commercial tau antibody reacting against the N-terminal end of tau. Dashes on the right side indicate the molecular weight (kDa) of bands calculated from migration of standard proteins.

(**B**) Histograms show the semi-quantitative densitometry of the intensity signals of bands (NH_2htau/total tau) by normalization with β-actin level used as loading control. $p<0.05$ was accepted as statistically significant (one-way ANOVA followed by Bonferroni’s post-hoc test for multiple comparison among more than two groups *$p<0.05$).

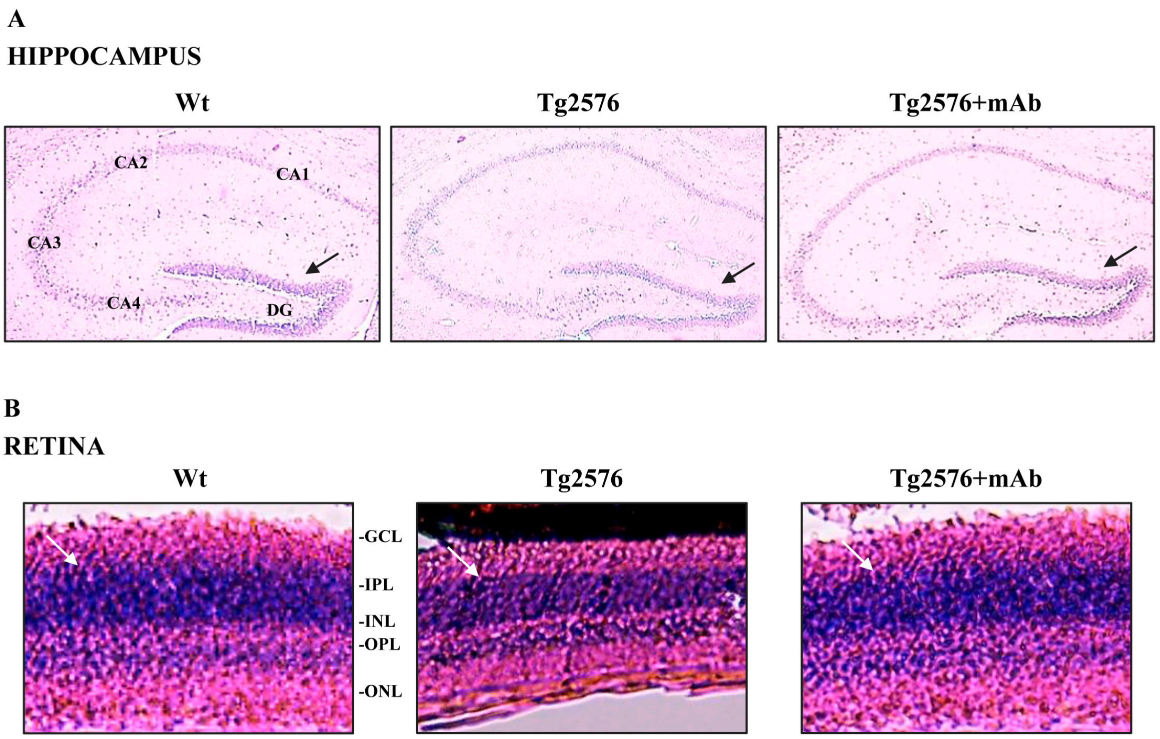

Supplementary Figure S3 Histopathological alterations in the hippocampus and retina from Tg2576 mice are mitigated following 12A12mAb-mediated neutralization of the NH_2htau.

(**A**,**B**) Representative images of hippocampal (**A**) and retinal (**B**) slices from three experimental groups (littermate wild-type, naive/vehicle-treated Tg2576, Tg2576+mAb) showing Haematoxylin and Eosin (H/E) staining under examination with a light electric microscope at 4X (n = 4 animals per each group, 2 males and 2 females for each experimental condition). Scale bar=50 m. Notice that hippocampal dentate gyrus (DG) sub-region and retinal stratification are reduced of thickness in Tg2576 mice when compared to wild-type controls whereas the overall tissue morphology is well-preserved following 12A12mAb administration (arrows).

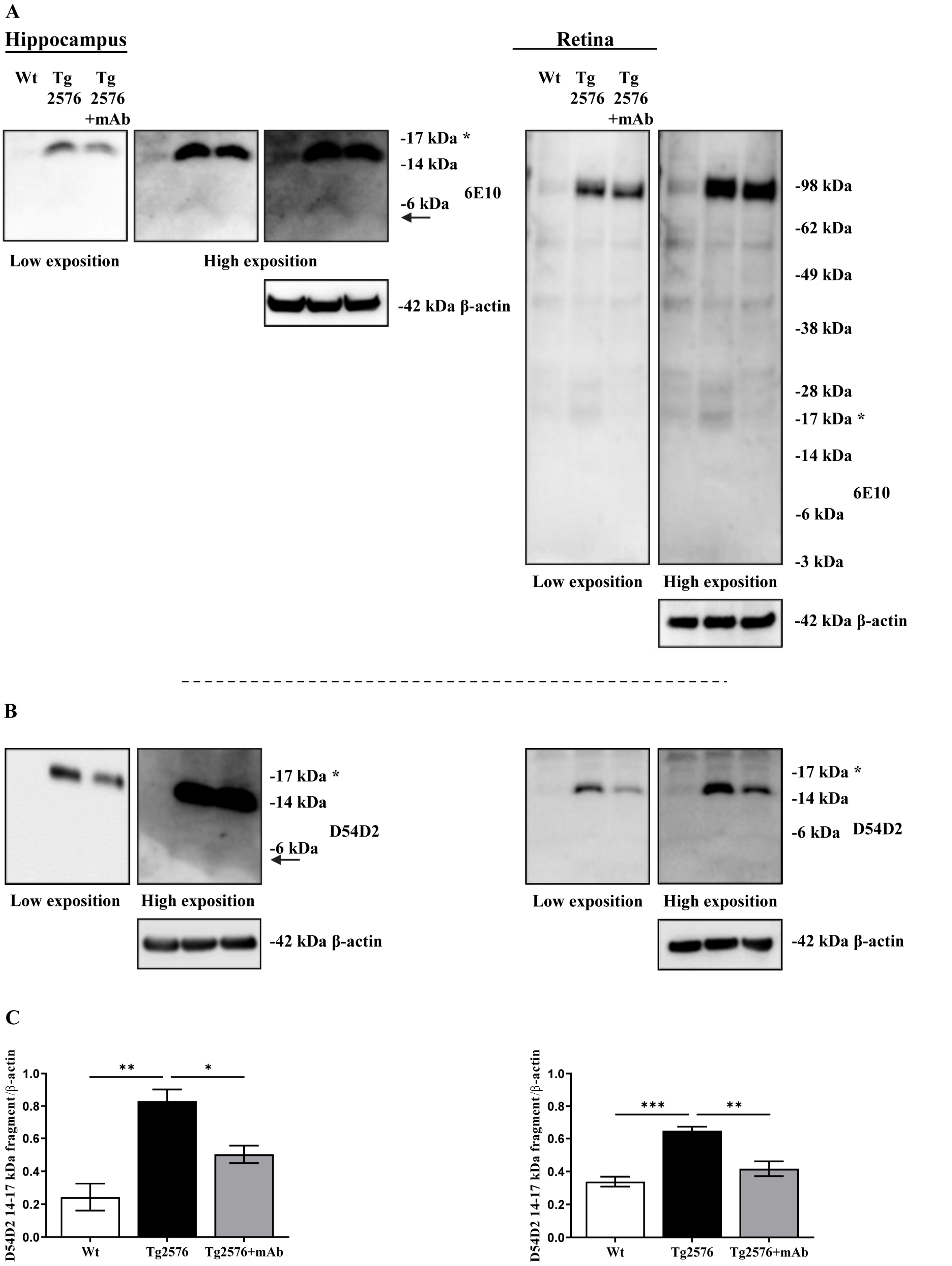

Supplementary Figure S4. The hippocampal and retinal amount of low-molecular weight (LMW) APP-derived A species is significantly reduced by i.v. delivery of 12A12mAb in Tg2576 AD mouse model.

(**A**) Representative images of SDS-PAGE Western blotting analysis (n = 6 animals per each group, 3 males and 3 females for each experimental condition) carried out on synaptosomal preparations of hippocampus (left) and retina (right) from animals of three experimental groups (littermate wild-type, naive/vehicle-treated Tg2576, Tg2576+mAb) with 6E10 antibody (reactive to aa 1-16 of Aβ and to APP). Dashes on the right side indicate the molecular weight (kDa) of bands calculated from migration of standard proteins. The arrow corresponds to the expression 4 kDa Aβ monomer whereas the asterisk corresponds to the expression of Aβ trimers and or β-CTFs derived from APP processing migrating around 14-17kDa. Notice that in retinal extracts of Tg2576 as well as in other mutated APP-overexpressing mouse strains, there is only a faint signal for 4 kDa Aβ peptide in agreement with the evidence that it is generated in the peripheral nervous system at a lower extent than in the brain [15].

(**B**) Representative images of SDS-PAGE Western blotting analysis (n = 6 animals per each group, 3 males and 3 females for each experimental condition) carried out on synaptosomal preparations of hippocampus (left) and retina (right) from animals of three experimental groups (littermate wild-type, naive/vehicle-treated Tg2576, Tg2576+mAb) with anti-β-Amyloid specific antibody (D54D2, Rabbit #8243 Cell Signaling).

(**C**) Histograms show the semi-quantitative densitometry of the intensity signals of 14-17 kDa D54D2-positive bands by normalization with β-actin level used as loading control. $p<0.05$ was accepted as statistically significant (one-way ANOVA followed by Bonferroni's post-hoc test for multiple comparison among more than two groups *$p<0.05$; **$p<0.01$; ***$p<0.0005$).

Supplementary Materials

Quality check of tissue homogenate preparations, containing mitochondria.

Mitochondrial analysis, as first step, requires the physically disruption of the cell membrane to release the cellular contents, maintaining the integrity and function of the mitochondria. For this purpose, the PBI-Shredder - an auxiliary high-resolution respirometry (HRR) Tool - was used to prepare homogenate of frozen tissue specimens, as reported in the Materials and Methods section. In order to verify both the complete rupture of the plasma membrane and the integrity of the mitochondrial ones, appropriate checks based on the measurement LDH activity [197] released from disrupted cells, as well as mitochondrial enzyme assays and respiratory experiments, used for evaluation of mitochondrial integrity and function, were made, respectively. LDH assay is a tool to estimate the mechanical rupture procedure of the cell membrane as a function of the amount of the LDH release in the medium [198]. Subsequent treatment with Triton-X-100 (0.2% in assay medium; at this concentration, Triton X-100 does not affect the LDH activity) did not cause an increase in activity, i.e. further release of LDH. The cell disruption efficiency is given by the relative LDH activity which was normalized to the activity measured in the disrupted cells by using 0.2% v/v Triton-x-100 (5 min incubation), corresponding to the maximum amount of releasable LDH enzyme activity [199]. Mitochondrial integrity was calculated on the basis of specific enzyme activity assays. Thus, the lack of activities of adenylate kinase (ADK, E.C.2.7.4.3) and glutamate dehydrogenase (GDH, E.C.1.4.1.3)[200] -which are markers of the mitochondrial intermembrane space and matrix, respectively- was monitored in the postmitochondrial supernatant obtained from cell homogenate after centrifugation at 10,000 rpm for 10 min at 4 °C to create a post-mitochondrial supernatant. For each sample, the free enzyme activity (as a result of damaged membranes) and the total enzyme

activity (total enzyme release was performed by chemical digestion with 0.2% T-x-100) were measured. The difference between total and free enzyme activity is referred to as latent activity. The ratio between latent and total enzyme activity is an index of the integrity of the mitochondrial membranes [201].

Further, the mitochondrial function, i.e. the coupling efficiency describing how much oxygen is needed to produce adenosine triphosphate (ATP), was checked by measuring the respiratory control index, i.e. (oxygen uptake rate after adenosine diphosphate [ADP] addition)/(oxygen uptake rate before ADP addition), which reflects the ability of mitochondria to produce ATP [200].
